# Perfluorophenyl-Incorporated Ferrocene: A Non-Volatile Solid Additive for Boosting Efficiency and Stability in Organic Solar Cells

**DOI:** 10.1021/acsami.5c04989

**Published:** 2025-05-20

**Authors:** Chia-Lin Tsai, Han-Cheng Lu, Chi-Chun Tseng, Yung-Jing Xue, Kai-En Hung, Chia-Shing Wu, Chia-Chih Chang, Chain-Shu Hsu, Katarina Gugujonovic, Markus Clark Scharber, Fong-Yi Cao, Yen-Ju Cheng

**Affiliations:** † Department of Applied Chemistry, 34914 National Yang Ming Chiao Tung University, 1001 University Road, Hsinchu 30010, Taiwan; ‡ Center for Emergent Functional Matter Science, National Yang Ming Chiao Tung University, 1001 University Road, Hsinchu 30010, Taiwan; § 124184 Taiwan Space Agency, 8F, 9 Prosperity 1st Road, Hsinchu Science Park, Hsinchu 300091, Taiwan; ∥ Department of Chemistry, 34910 National Changhua University of Education, Changhua City 50007, Taiwan; ⊥ Institute of Physical Chemistry and Linz Institute of Organic Solar Cells (LIOS), 27266 Johannes Kepler University Linz, Altenbergerstrasse 69, 4040 Linz, Austria

**Keywords:** solid additives, nonfullerene acceptors, morphology control, organic solar cells, non-volatile additives, perfluorophenyl derivatives

## Abstract

In this study, we designed and synthesized a new non-volatile solid additive FcF_10_ by integrating two pentafluorophenyl (C_6_F_5_) groups into the cyclopentadienyl (CP) rings of ferrocene (Fc) through ester linkages. The FcF_10_ with a three-dimensional (3D) framework facilitated morphological optimization in the PM6:Y6 system through a combination of π···π, F···π, and F···F interactions between the CP and C_6_F_5_ rings in FcF_10_ and the C_6_F_2_ rings in Y6. The FcF_10_-incorporated (3.75 wt %) PM6:Y6-based solar cell device achieved a higher power conversion efficiency (PCE) of 17.00%, with a *V*_oc_ of 0.85 V, a *J*_sc_ of 27.35 mA cm^–2^, and an FF of 73.29%, compared to the pristine PM6:Y6 device. These improvements are attributed to the formation of a favorable active layer morphology, which enhances exciton dissociation and charge transport while reducing bimolecular and trap-assisted recombination. The FcF_10_ additive facilitates non-covalent interactions with Y6, such as F···F, F···π, and π···π interactions between the Cp and C_6_F_5_ rings in FcF_10_ and the FIC end groups in Y6. These supramolecular interactions improve molecular stacking and crystallinity within the Y6 domain, as evidenced by red-shifted Y6 absorption, reduced π–π stacking *d*-spacing, and increased coherence lengths of Y6. Furthermore, the PM6:Y6:FcF_10_ device demonstrates superior thermal stability, retaining 88% of its initial PCE after prolonged thermal annealing at 85 °C. Overall, the incorporation of FcF_10_ achieves an optimized and stable donor–acceptor morphology, offering a promising approach for high-performance and thermally stable organic photovoltaics.

## Graphical abstract

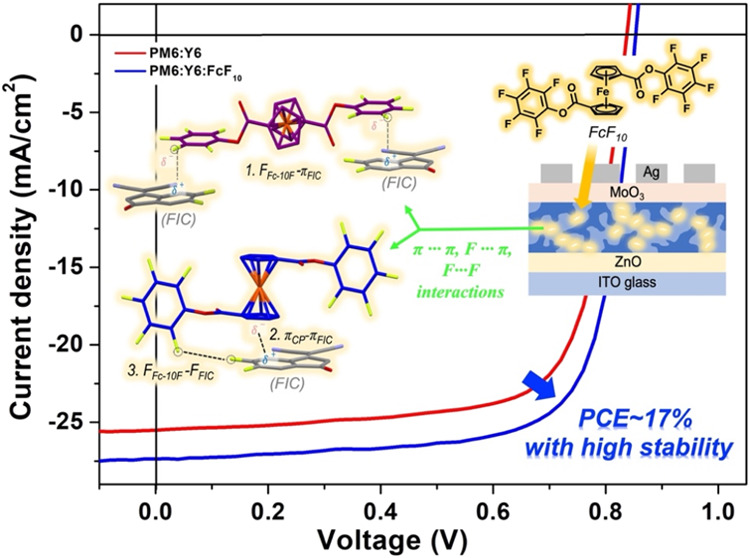

## Introduction

Organic solar cells (OSCs) have gained significant attention as promising solar energy converters due to their lightweight nature, low production cost, mechanical flexibility, and potential for large-area fabrication.^1–11^ To achieve commercial viability, it is essential to combine high power conversion efficiency (PCE) with long-term operational stability. Recent advancements in Y-series nonfullerene acceptors (NFAs) have propelled OSCs to PCEs exceeding 19% when paired with compatible donor materials.^12–21^ These breakthroughs underscore the practical potential of OSCs in energy applications. Alongside the development of new active materials, morphology control emerges as a pivotal strategy for further optimizing both the PCE and device stability. Bulk heterojunction (BHJ) devices, characterized by an optimal donor–acceptor interfacial area, are widely adopted for their ability to facilitate efficient charge separation. Achieving the desired morphology in the active layer has been a primary focus of research, with extensive efforts devoted to microstructure regulation. Among the various factors influencing blend nanostructures, intrinsic intermolecular interactions between donor and acceptor molecules, as well as their relative solubility in the processing solvent play a critical role. Various approaches have been developed to manipulate phase separation and domain behavior in active layers, including multi-component blending systems, thermal/solvent annealing,^22–25^ and additive treatments.^26–33^ Among these, the incorporation of additives has emerged as an effective and cost-efficient strategy for enhancing the performance of organic solar cells.

Liquid additives, such as 1,8-diiodooctane (DIO), diphenyl ether (DPE), and 1-chloronaphthalene (CN), are widely used due to their high boiling points and selective solubility, which enable precise control over donor–acceptor phase separation and the formation of an optimized bicontinuous network. However, the high boiling points can leave residual traces in the active layer, leading to potential morphological degradation, reduced reproducibility, and compromised stability.^34–37^ Solid additives, a promising alternative to liquid additives, can be categorized into two types based on their presence in the active layer: volatile solid additives and non-volatile solid additives. Unlike liquid additives, solid additives can effectively stabilize the morphology of the active layer, thereby enhancing the device stability. Their working mechanisms include strengthening donor–acceptor interactions, such as hydrogen bonding,^38,39^ π–π stacking, halogen bonding, and quadrupole moment interactions.^40^ They also inhibit crystal nucleation and growth,^41,42^ reduce intermolecular adsorption energy,^43,44^ and increase the free volume within donor and/or acceptor materials.^45^ In recent years, various volatile solid additives have been described to enhance the efficiency and stability of OSCs, leading to significant advancements in device performance. The function of volatile solid additives is to improve active layer bicontinuous morphology by enhancing intermolecular π–π interactions and crystallinity of polymers and NFA nanodomains, leading to enhanced exciton dissociation and charge transfer.^46–50^ In 2020, Sun and co-workers introduced ferrocene (Fc) as a volatile solid additive in PM6:Y6 blends, achieving a PCE of 17.4%, which significantly surpassed the 15.5% PCE of devices without additives and the 16.5% PCE achieved with the CN solvent additive.^51^ The enhanced efficiency with ferrocene was attributed to increased molecular crystallinity, improved charge transport, and reduced rate of charge recombination. Ferrocene-treated OSCs also demonstrated superior photostability compared with CN-processed devices. Compared to volatile additives, non-volatile additives have demonstrated multifunctional roles in OSCs, acting as molecular locks, morphological controllers, and stacking promoters.^52^ These additives stabilize the active layer morphology, suppress phase separation, improve thermal stability, and enhance both device performance and fabrication reproducibility. We recently developed a non-volatile additive, bis­(perfluorophenyl)­pimelate (BF7), featuring two perfluorophenyl (C_6_F_5_) groups linked to a linear aliphatic chain via ester linkages. The hand-grab-like C_6_F_5_ moieties of BF7, retained in the film after processing, form supramolecular physical attractions with fullerene derivatives, suppressing their aggregation.^53^ As a result, [6,6]-phenyl-C_61_-butyric acid methyl ester-based solar cells with BF7 incorporation demonstrated not only improved efficiency but also exceptional thermal stability.^54^ For Y6-based OSCs, PM6:Y6:BF7 devices achieved a PCE of 17.01%, compared to 15.16% for those without BF7. Moreover, the BF7-enhanced PCE maintained 95% of its peak performance after 72 h of annealing at 100°C. This improvement arises from preferential F−π supramolecular interactions between the C_6_F_5_ moieties of BF7 and the difluorophenyl (C_6_F_2_)-based FIC end groups of Y6. These interactions optimize the active layer morphology by creating fractal-like network structures with ideal Y6 nanocrystallite size and orientation, leading to enhanced thermal stability.^55^

In this study, we combined the advantages of Fc and BF7 by designing a new non-volatile solid additive, FcF_10_, which was synthesized by integrating two pentafluorophenyl (C_6_F_5_) groups into the cyclopentadienyl (CP) rings of Fc through ester linkages. The FcF_10_ with the three-dimensional (3D) framework facilitated morphological optimization in the PM6:Y6 system through a combination of π···π, F···π, and F···F interactions between the CP and C_6_F_5_ rings in FcF_10_ and the C_6_F_2_ rings in Y6, leading to a significant enhancement in device performance. The FcF_10_-incorporated (3.75 wt %) PM6:Y6 device achieved a PCE increase from 15.34 to 17.00%, driven by an improved fill factor (FF) of 73.29% and a short-circuit current density (*J*_sc_) of 27.35 mA/cm^2^. Ultraviolet–visible (UV–Vis) spectroscopy, space-charge-limited current (SCLC) measurements, charge recombination analysis, atomic force microscopy (AFM), and grazing-incidence wide-angle X-ray scattering (GIWAXS) have been utilized to investigate the morphological and electrical effects introduced by FcF_10_.

## Experimental Section

### Synthesis of FcF_10_

A solution of thionyl chloride (0.12 mL) in dichloromethane (2 mL) was added dropwise to a solution of 1,1′-ferrocenedicarboxylic acid (200.0 mg, 0.73 mmol) of freshly dried dichloromethane (3 mL) at 0 ^o^C. The solution was first stirred for 2 h at 0 ^o^C and then for 6 h at room temperature. After the completion of the reaction, solvent was evaporated to give the intermediate 1,1′-ferrocenedicarbonyl chloride without purification. A solution of pentafluorobenzyl alcohol (295.5 mg, 1.61 mmol) and triethylamine (0.24 mL) in dichloroethane (2 mL) was added to the acid chloride solution in dichloroethane (3 mL). The reaction mixture was refluxed for 12 h. The product was extracted with dichloromethane and water for 3 times. The organic collection layer was dried over anhydrous MgSO_4_. After the solvent was removed under reduced pressure, the residue was purified by column chromatography on silica gel (dichloromethane/hexane 1/3) to afford an orange solid of 199 mg (45%). ^1^H NMR (400 MHz, CDCl_3_) δ 5.11 (m, 4H), 4.73 (m, 4H). ^13^C NMR (100 MHz, CDCl_3_) δ: 166.64, 141.34, 139.54, 137.97, 125.02, 74.99, 72.80, 69.17. HRMS (FD, C_24_H_8_O_4_F_10_Fe): calcd, 605.9617; found 605.9607.

## Results and Discussion

The synthesis of FcF_10_ is depicted in Scheme 1. 1,1′-Ferrocenedicarboxylic acid was reacted with thionyl chloride to form a Fc-COCl intermediate, which was immediately reacted with 2,3,4,5,6-pentafluorophenol to yield FcF_10_ in a 45% yield. The detailed synthetic procedure, mass spectrometry, and ^1^H NMR and ^13^C NMR of new compounds are shown in the Experimental Section and Supporting Information.

**Scheme 1:**
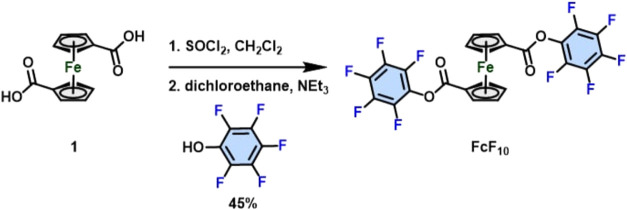
Synthesis of FcF_10_

UV–Vis absorption spectra were employed to illustrate the effect of intermolecular interactions upon addition of FcF_10_ to PM6 and Y6. Figure 1 displays the normalized solid-state absorption spectra of pristine PM6 and Y6 films, both with and without 3.75 wt % FcF_10_ additive. Additionally, we compared the absorption spectra before and after thermal annealing at 100°C. In the case of PM6, the pristine thin film exhibited a λ_max_ at 612 nm, with no significant shifts observed after adding FcF_10_ (614 nm). After thermal annealing, the spectra showed no notable change compared with those without annealing, indicating that FcF_10_ did not affect the donor domain. Conversely, the Y6 film showed a red shift in λ_max_ from 808 to 819 nm and a decreased shoulder at 750 nm upon the addition of FcF_10_. After thermal annealing, the λ_max_ further red-shifted by 8 to 827 nm. These absorption spectra results suggest that the addition of FcF_10_ might promote increased *J*-aggregation in Y6 with tightened molecular packing.

**Figure 1:**
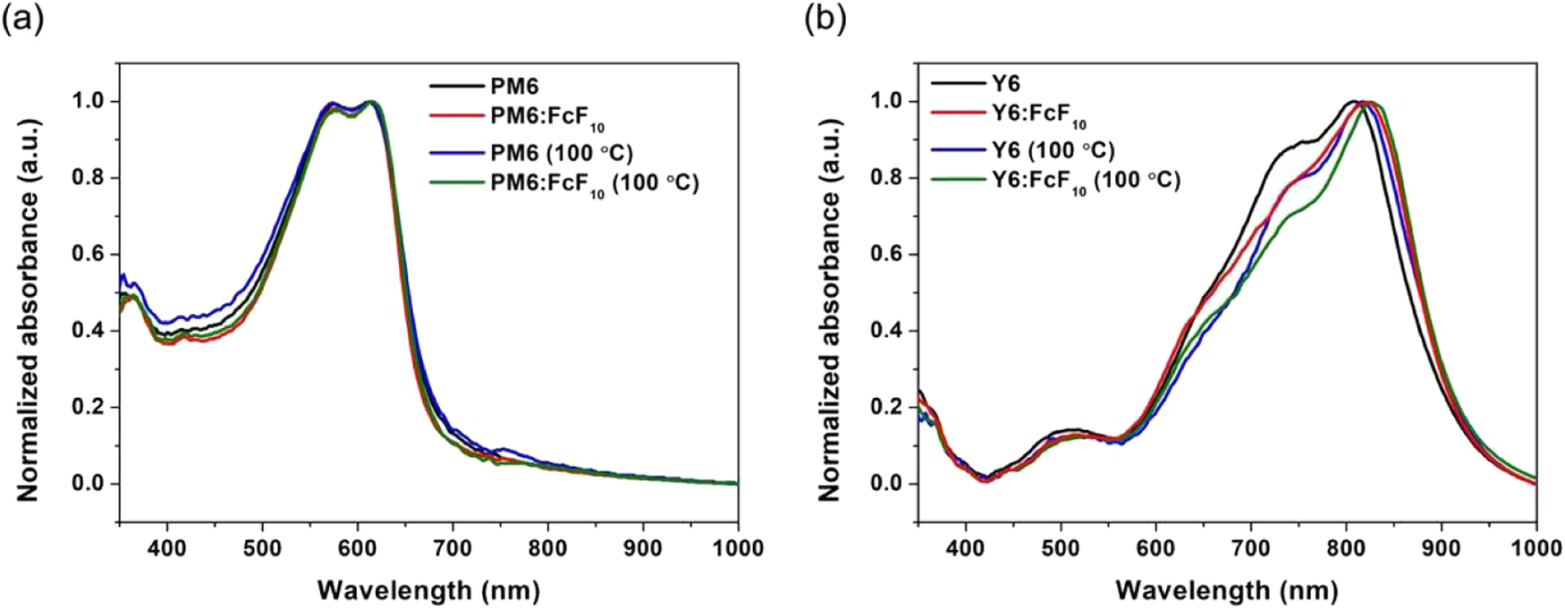
UV–Vis absorption spectra of (a) PM6 and (b) Y6 thin films with and without adding FcF_10_ and thermal annealing treatment at 100 °C.

Thermal properties were evaluated by using thermogravimetric analysis (TGA) and differential scanning calorimetry (DSC). First, we measured the weight loss of FcF_10_ under increasing temperatures with a ramp rate of 10 °C/min, as shown in Figure 2a. The results indicate that the weight of FcF_10_ begins to decrease at 137 °C, suggesting the onset of sublimation. Additionally, DSC measurements in Figure 2b revealed a strong endothermic peak at 137 °C, consistent with the TGA results. To confirm that FcF_10_ acts as a non-volatile solid additive in the device processing, we conducted an isothermal measurement at 100 °C (the thermal annealing temperature). After 1 h at 100 °C, FcF_10_ showed nearly no weight loss, implying its stability under thermal annealing conditions.

**Figure 2:**
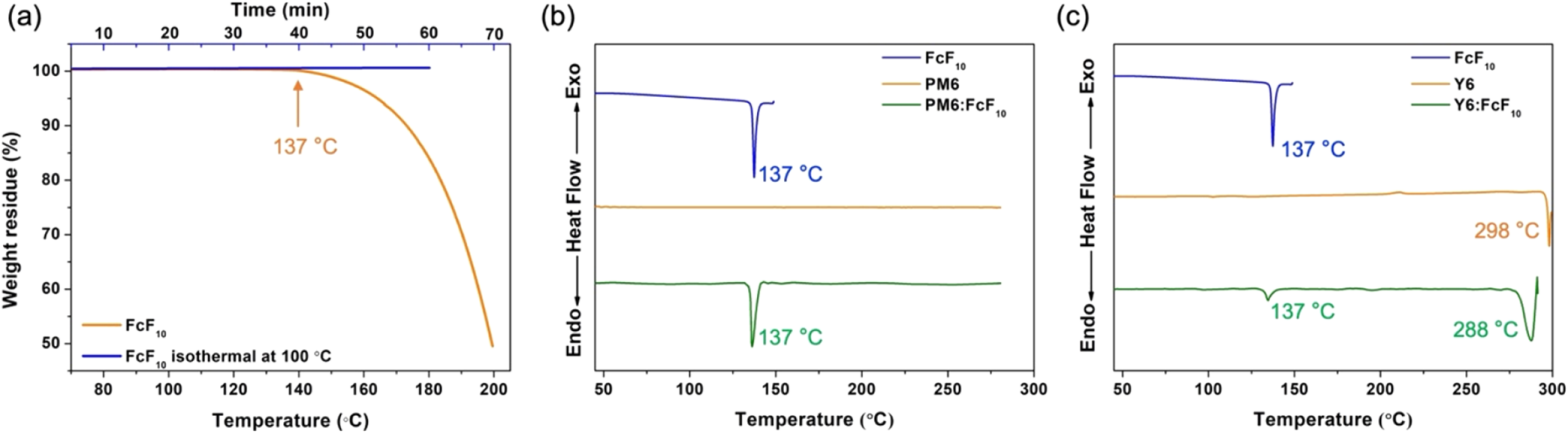
(a) TGA and isothermal measurements at 100 °C of FcF_10_. DSC measurements of (b) FcF_10_, pristine PM6, and PM6 with FcF_10_; (c) FcF_10_, pristine Y6, and Y6 with FcF_10_ with a ramping rate of 10 °C/min.

To further investigate the effect of FcF_10_ on the morphology of PM6 and Y6, we blended FcF_10_ (3.75 wt %) into the polymer donor PM6 or acceptor Y6. As shown in Figure 2c, we observed that PM6 with FcF_10_ exhibit only a weak endothermic peak at 137 °C, corresponding to the sublimation temperature of FcF_10_. This observation suggests that FcF_10_ does not significantly affect the morphology of the PM6 domain. In contrast, Figure 2c shows that the melting temperature (*T*_m_) of Y6 decreased from 298 to 288 °C upon the addition of FcF_10_, while the characteristic peak of FcF_10_ remained unchanged. These results suggest that FcF_10_ primarily influences the stacking and interactions among the Y6 molecules. It is speculated that FcF_10_ tends to localize at the interfaces between Y6-rich domains, acting as a three-dimensional spacer that weakens inter-domain interactions and leads to a slightly lower *T*_m_.

To investigate the photovoltaic properties of devices incorporating FcF_10_ as an additive, inverted configuration OSCs with an ITO/ZnO/active layer/MoO_3_/Ag architecture were fabricated using PM6 and Y6 as the active layer materials. Detailed fabrication methods are described in the Supporting Information. The characteristics, *J–V* curves, and external quantum efficiency (EQE) spectra of these devices are presented in Table 1 and Figure 3. The reference device, consisting of a PM6:Y6 blend with 0.6 vol % of CN, exhibited a *V*_oc_ of 0.84 V, a *J*_sc_ of 25.50 mA cm^–2^, and an FF of 71.74%, resulting in a PCE of 15.34%, consistent with values reported in the literature. To optimize FcF_10_ content, its weight ratio was screened from 0.625 to 5 wt % relative to PM6, with the device performance summarized in Table S1. Compared to the reference device, the optimized device with 3.75 wt % of FcF_10_ showed a slightly higher *V*_oc_ of 0.85 V, notable increase in *J*_sc_ to 27.35 mA cm^–2^, and a slight improvement in FF to 73.29%, enhancing the PCE to 17.00%. The comparable *V*_oc_ values between devices with and without FcF_10_ suggest that FcF_10_ does not alter the energy levels of PM6 or Y6. The observed improvements in *J*_sc_ and FF are primarily attributed to enhanced molecular ordering, improved charge transport, and reduced recombination rather than changes in energy level offsets. Further EQE measurements, shown in Figure 3b, indicated higher EQE values around 650 and 830 nm regions, aligning with the maximum characteristic absorption regions of PM6 and Y6. This enhancement suggests that FcF_10_ improves the PM6 and Y6 domains and optimizes charge carrier kinetics, leading to concurrent improvements in *J*_sc_ and FF. Additionally, the red-shifted absorption spectrum of the FcF_10_-modified PM6 blend broadens the absorption range, contributing to higher carrier generation and improved *J*_sc_. To further investigate charge transport, hole-only devices (ITO/PEDOT/active layer/Au) and electron-only devices (Al/active layer/Al) were fabricated to evaluate hole and electron mobilities using the space-charge-limited current (SCLC) model (Figure S1). As shown in Figure S1 and Table 1, the hole and electron mobilities for the pristine PM6:Y6 blend were estimated at 1.12 × 10^–4^ cm^2^ V^–1^ s^–1^ and 1.34 × 10^–4^ cm^2^ V^–1^ s^–1^, respectively. In contrast, the PM6:Y6 blend with FcF_10_ exhibited enhanced mobilities of 2.14 × 10^–4^ cm^2^ V^–1^ s^–1^ for holes and 2.53 × 10^–4^ cm^2^ V^–1^ s^–1^ for electrons. The improvement in hole and electron mobilities in the FcF_10_-modified blend suggests that the inclusion of FcF_10_ enhances intermolecular stacking within the p- and n-type domains, facilitating charge carrier transport. This improvement in mobility is consistent with the observed increases in *J*_sc_ and FF in the devices, as it enables more efficient charge extraction and reduced recombination.

**Figure 3:**
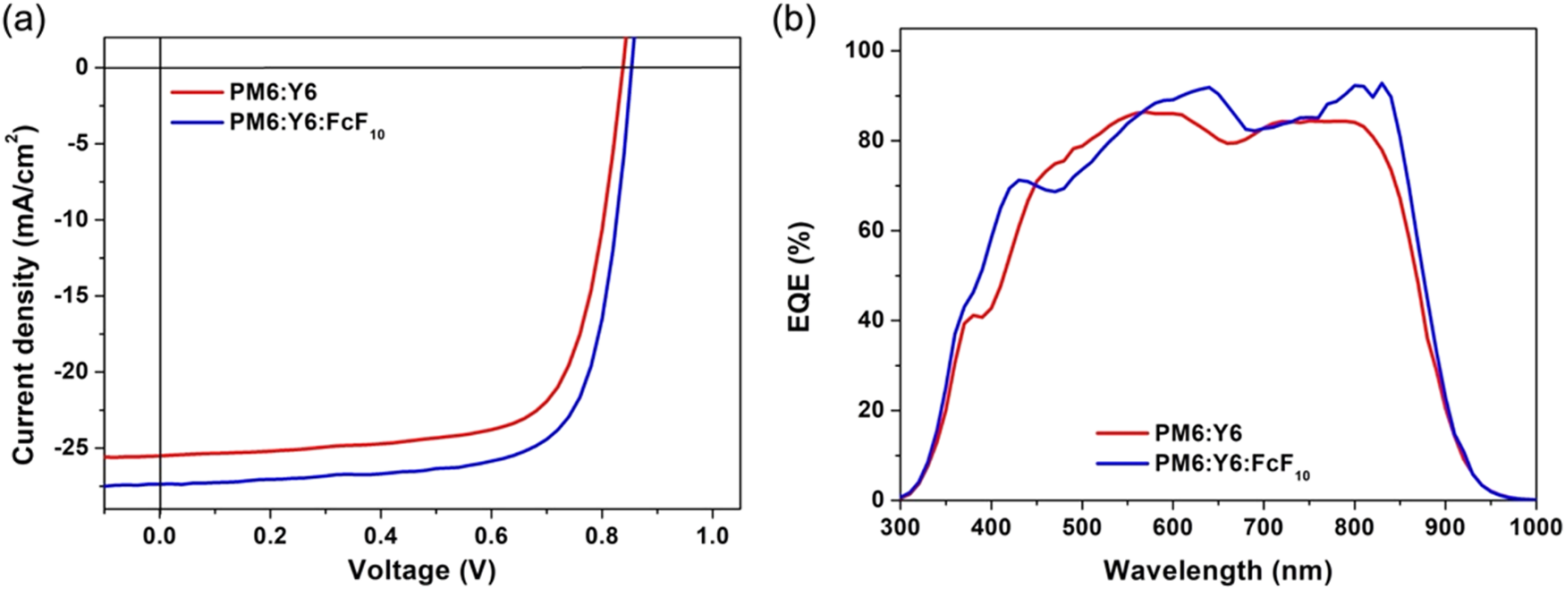
(a) *J*–*V* curves and (b) EQE spectra of PM6:Y6-based devices with and without FcF_10_.

**Table 1:** Characteristics of PM6:Y6-Based Devices with and without Optimized FcF_10_ Weight Content^a^

FcF_10_ (wt %)	*V*_oc_ (V)	*J*_sc_ (mA/cm^2^)	FF (%)	PCE (%)	μ_h_ (cm^2^ V^–1^ s^–1^)	μ_e_ (cm^2^ V^–1^ s^–1^)	μ_h_/μ_e_ ratio
NA	0.84 (0.83 ± 0.01)	25.50 (25.42)^b^ (25.84 ± 0.60)	71.74 (70.50 ± 1.61)	15.34 (15.18 ± 0.21)	1.12 × 10^–4^	1.34 × 10^–4^	0.84
3.75	0.85 (0.85 ± 0.01)	27.35 (26.72)^b^(26.87 ± 0.43)	73.29 (72.63 ± 0.86)	17.00 (16.53 ± 0.31)	2.14 × 10^–4^	2.53 × 10^–4^	0.85

a The average value was calculated from 10 devices.

b *J*_sc_ calculated by EQE. μ_h_ = hole mobility, μ_e_ = electron mobility.

To investigate the influence of FcF_10_ on charge recombination in the device, the dependence of *J*_sc_ and *V*_oc_ on light intensity (*P*_light_) was studied. The relationship between *J*_sc_ and *P*_light_ can be described by the power-law equation *J*_sc_ ∝ (*P*_light_)^α^, where α indicates the degree of bimolecular recombination. As shown in Figure 4a, the extracted α values for devices without and with FcF_10_ were 0.947 and 0.973, respectively, indicating that bimolecular recombination was effectively reduced in the FcF_10_-processed device under short-circuit conditions. Furthermore, the relationship between *V*_oc_ and *P*_light_ helps analyze trap-assisted recombination within the devices. By the examination of the slope of *V*_oc_ versus the natural logarithm of *P*_light_, the recombination mechanism can be clarified. As shown in Figure 4b, OSCs with only the CN additive exhibited a slope of 1.19 kT/q, while devices with FcF_10_ as an additive had a slope of 1.08 kT/q. These results suggest that the use of FcF_10_ effectively suppresses trap-assisted recombination, contributing to improved device performance.

**Figure 4:**
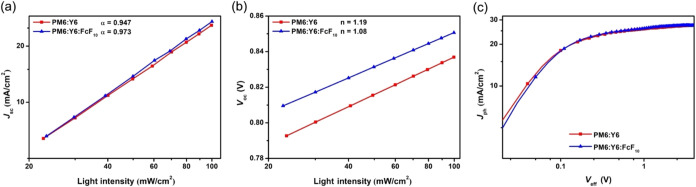
Plots of (a) dependences of *J*_sc_ and (b) *V*_oc_ on light intensity *P*_light_, and (c) *J*_ph_ against *V*_eff_ for the PM6:Y6-based devices with or without FcF_10_.

To investigate the factors contributing to the enhanced *J*_sc_ and FF, measurements of the saturation current density *J*_sat_ and exciton dissociation probability (*η*_diss_) of optimized devices were performed. The photocurrent density (*J*_ph_) versus effective voltage (*V*_eff_) curves are shown in Figure 4c. Here, *J*_ph_ represents the difference between *J*_L_ and *J*_D_, where *J*_L_ and *J*_D_ denote the current density under illumination and in the dark, respectively. *V*_eff_ is defined as the difference between *V*_0_ (the voltage at which *J*_ph_ is zero) and *V*_app_ (the applied bias voltage). It was observed that *J*_ph_ reached *J*_sat_ when *V*_eff_ approached 2 V, suggesting that all of the excitons in the device were dissociated into free carriers and collected by the electrodes. The *J*_sat_ values for devices without and with FcF_10_ were 27.13 and 27.34 mA cm^–2^, respectively, indicating that FcF_10_ enhances the photon harvesting capability of the PM6:Y6 blend. Under short-circuit conditions, PM6:Y6 and PM6:Y6:FcF_10_ devices exhibited *η*_diss_ values of 95.28 and 95.97%, respectively, and charge collection probabilities (*η*_coll_) of 80.39 and 81.91%, respectively. These results demonstrate that FcF_10_ aids in charge extraction, contributing to the observed improvements in *J*_sc_ and FF.

Single-crystal structures and crystallographic data of FcF_10_ are shown in Figure 5a and Table S2. The detailed single-crystal measurement is included in the Supporting Information. It is interesting to observe that the planes of two C_6_F_5_ rings are oriented almost perpendicularly to the plane of two CP rings in a FcF_10_ molecule. A unit cell in the single-crystal structure consists of three FcF_10_ molecules that self-assemble into two types of center-offset plane-to-plane π–p stacking modes. In the first mode, the C_6_F_5_ ring of one FcF_10_ molecule (purple) aligns parallel in center-offset stacking with another C_6_F_5_ ring of an adjacent FcF_10_ molecule (black). This interaction is reinforced by the electronegative peripheral F atoms of the C_6_F_5_ ring interacting with the electropositive center of the adjacent C_6_F_5_ ring (*F*_FcF10_–π_FcF10_) with a plane-to-plane distance of 3.502 Å. In the second arrangement, regarded as the π_FcF10_–π_CP_ mode, the electropositive center of the C_6_F_5_ ring of FcF_10_ (purple) aligns parallel and center-offset with the electronegative center of the CP ring of an adjacent FcF_10_ molecule (blue) with a shorter plane-to-plane distance of 3.486 Å.

**Figure 5:**
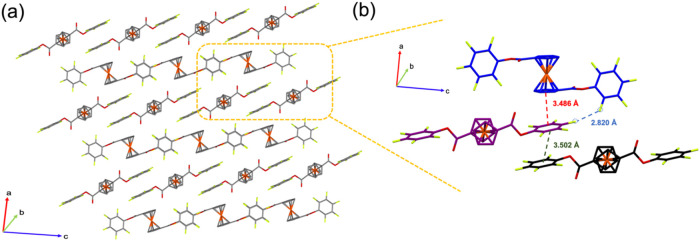
(a) Single-crystal structure and intermolecular packing of FcF_10_. (b) Molecular packing in a unit cell of FcF_10_, showing two center-offset stacking modes via π···π, F···π, and F···F interactions.

Additionally, Figure 5b illustrates that the fluorine atom on the *para* position of the C_6_F_5_ ring of the central FcF_10_ molecule (purple) engages in F···F interactions with the fluorine atom on the *ortho* position of adjacent molecules (blue) (*para-*F-*ortho*-F). This F···F interaction, with a short contact of 2.820 Å, aligns the molecules directionally, causing the planes of the CP (blue) and PFB (purple) rings to be slightly non-parallel.

Similarly, we propose that the terminal difluorophenyl moeity of FIC, the end group of Y6, can form analogous molecular interactions with FcF_10_, including interactions between (1) the electronegative F atom of FcF_10_ and the electropositive difluorophenyl ring of FIC (*F*_FcF10_ – π_FIC_) and (2) vice versa (*F*_FIC_ – π_FcF10_), (3) the Cp ring of FcF_10_ and the difluorophenyl ring of FIC (π_CP_ – π_FIC_), and (4) F atoms on FcF_10_ and FIC (*F*_FcF10_ – *F*_FIC_). These intermolecular interactions could not only optimize nanocrystalline domain formation but also stabilize the morphology, enhancing the device stability.

The intermolecular π–π stacking distances and nanocrystalline domain sizes of molecular arrangements in thin films were evaluated by using grazing-incidence wide-angle X-ray scattering (GIWAXS). We systematically investigated three systems: PM6 and Y6 neat films, the PM6:Y6 blend films, and their FcF_10_-incorporated counterparts. A summary of the GIWAXS data and related details is provided in Table 2. The GIWAXS two-dimensional (2D) image of the neat PM6 film displays a diffraction peak at *q*_*z*_ =1.75 Å^–1^ along the out-of-plane direction, corresponding to PM6’s π–p stacking *d*-spacing of 3.59 Å (Figure 6a). After the addition of FcF_10_, the peak at *q*_*z*_ =1.75 Å^–1^ remains unchanged in the PM6:FcF_10_ blend (Figure 6b). However, the peak becomes narrower and more intense, indicating FcF_10_ induces an increase in coherence length (CL) of PM6 π–π stacking domain from 22.32 to 24.12 Å. For Y6 (Figure 6c,6d), the incorporation of FcF_10_ shifted the π–π stacking signal peak in the out-of-plane direction from *q*_*z*_ = 1.802 to 1.812 Å^–1^, corresponding to a decrease in the π–p stacking d-spacing from 3.49 to 3.45 Å. Moreover, the CL of Y6 increased from 24.09 to 25.16 Å, indicating improved crystallinity and the formation of larger crystal grains upon FcF_10_ addition. The closer stacking of Y6 molecules might be ascribed to the non-covalent interactions such as F···F, F···π, and π···π interactions between Y6 and FcF_10_. The selective effect of FcF_10_ on Y6 rather than on PM6 arises from the chemical structure of the two materials. Y6 contains terminal difluorobenzene (FIC) units capable of engaging in strong F···F, F···π, and π–π interactions with the perfluorinated moieties of FcF_10_. In contrast, PM6 lacks such reactive end groups, resulting in minimal interaction with FcF_10_ and negligible influence on its stacking behavior. For the PM6:Y6 blend system shown in Figure 6e,6f, the active layer showed an increase in CL from 19.13 to 20.94 Å upon FcF_10_ addition, suggesting larger molecular grain sizes. Notably, the π–π stacking and d-spacing of the PM6:Y6 blend remained constant at 3.53 Å. The corresponding one-dimensional (1D) line-cut profiles of GIWAXS diffraction patterns of these thin films are shown in Figure S2. This improved structural arrangement likely facilitates carrier mobility by minimizing carrier scattering at grain boundaries, aligning with the observed increases in electron and hole mobility from space-charge-limited current (SCLC) measurements.

**Figure 6:**
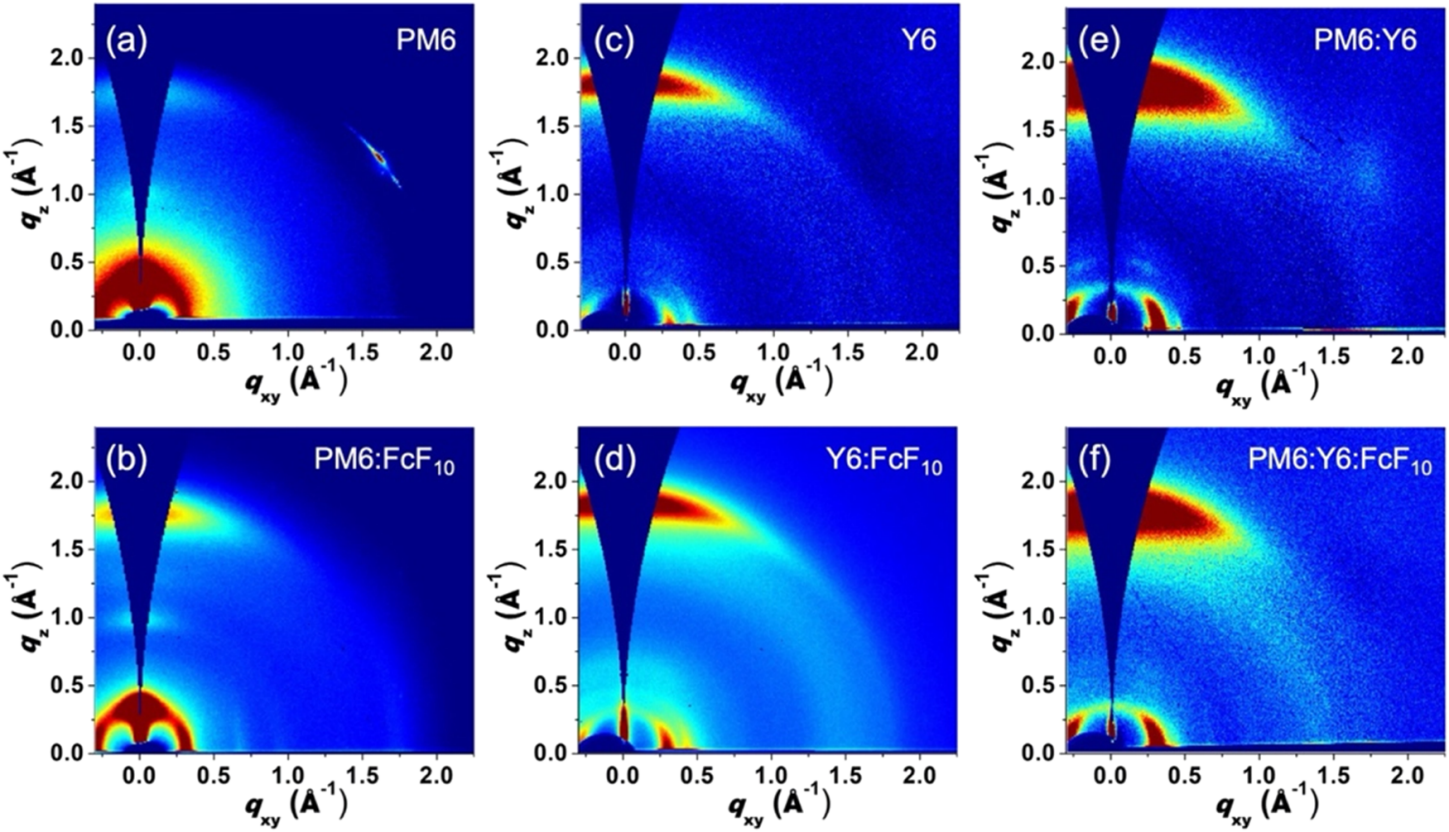
2D GIWAXS patterns of the thin films of (a) PM6, (b) PM6:FcF_10_, (c) Y6, (d) Y6:FcF_10_, (e) PM6:Y6, and (f) PM6:Y6:FcF_10_.

**Table 2:** GIWAXS Data of PM6, Y6, PM6:Y6, and the FcF_10_-Incorporated Counterparts

	in plane		out of plane			
materials	*q*_*xy*_ (Å^–1^)	*d*-spacing^a^ (Å)	*q*_*z*_ (Å^–1^)	*d*-spacing^a^ (Å)	FWHM (Å^–1^)	CL^b^ (Å)
PM6	0.28	22.39	1.75	3.59	0.25	22.32
PM6:FcF_10_	0.29	21.59	1.75	3.59	0.23	24.12
Y6	0.29	22.00	1.80	3.49	0.24	24.09
Y6:FcF_10_	0.29	22.00	1.82	3.45	0.23	25.16
PM6:Y6	0.30	21.00	1.78	3.53	0.30	19.13
PM6:Y6:FcF_10_	0.29	21.48	1.78	3.53	0.27	20.94

a *d*-spacing = 2π/q.

b CL =2 kπ/q.

The surface morphology of the active layers was examined using atomic force microscopy (AFM), as illustrated in Figure 7, which presents AFM height images of pristine Y6 and PM6:Y6 blend films along with their FcF_10_-modified counterparts. The incorporation of FcF_10_ resulted in an increase in surface root-mean-square roughness (*R*_q_) from 2.46 to 3.85 nm for the Y6 film. The notable increase in *R*_q_ for the Y6 film corresponds to enhanced crystallinity, as evidenced by the increased CL observed in the GIWAXS analysis. This phenomenon suggests that intermolecular interactions induced by FcF_10_, such as *F*_FcF10_···π_FIC_, π_CP_···π_FIC_, and *F*_FcF10_···*F*_FIC_ have a greater influence on the molecular stacking of Y6. This observation aligns with the DSC and UV–Vis spectroscopy results. Additionally, the PM6:Y6 blend system exhibited an increase in *R*_q_ from 2.37 to 3.15 nm upon FcF_10_ addition, suggesting that FcF_10_ promotes a more favorable morphology for phase separation.

**Figure 7:**
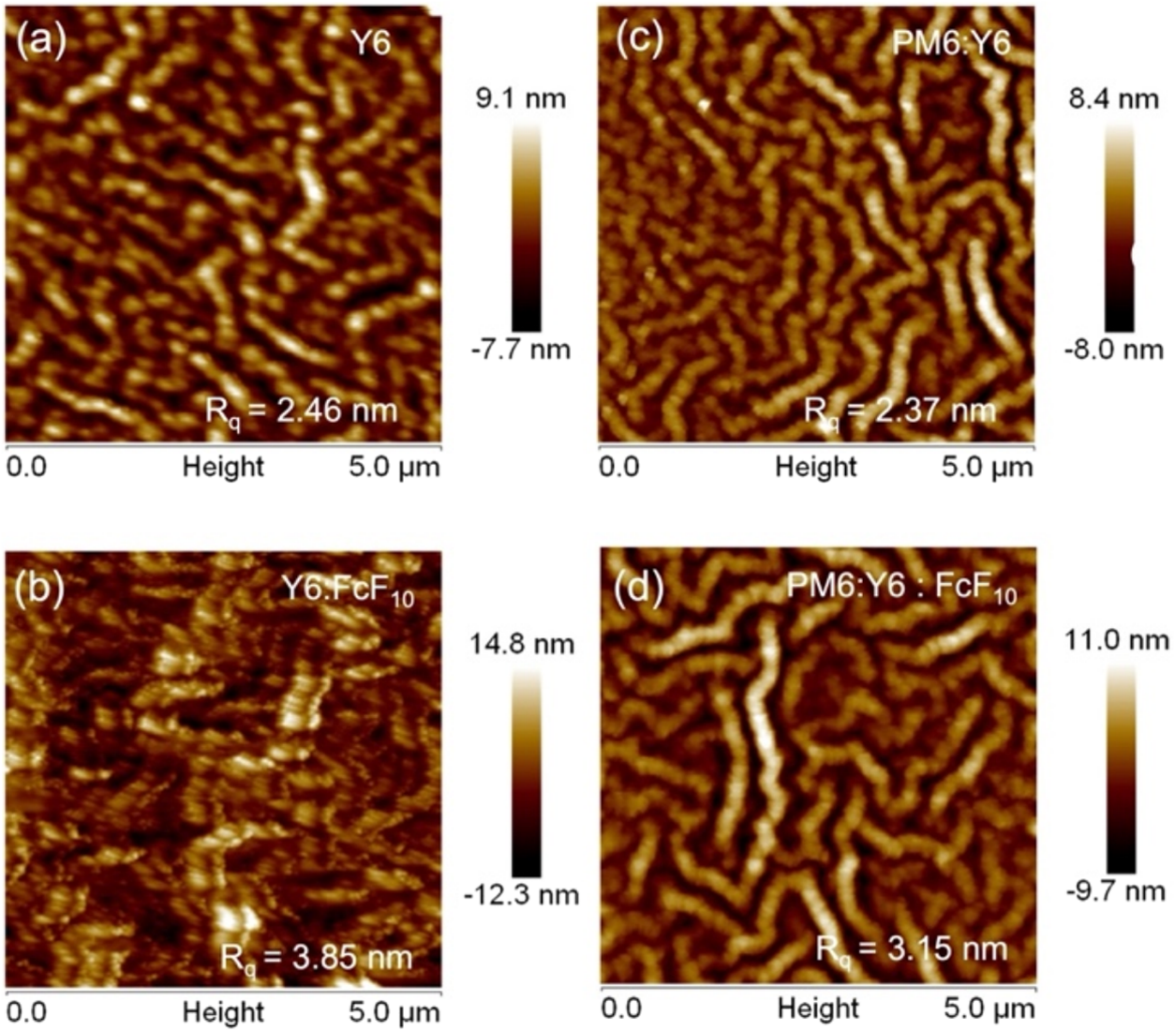
AFM height images of the (a) Y6, (b) Y6:FcF_10_, (c) PM6:Y6, and (d) PM6:Y6:FcF_10_ thin films.

The thermal stability of the devices was evaluated by monitoring their performance upon storage at 85 °C, addressing the high-temperature challenges typically faced by OSCs during prolonged exposure. As shown in Figure 8, after prolonged annealing at 85 °C for 360 h, the PM6:Y6 device with FcF_10_ demonstrated superior thermal stability, retaining 88% of its initial PCE compared to 81% for the device without FcF_10_. These findings indicate that FcF_10_ not only enhances the performance but also strengthens the device stability. This improvement is associated with the preferential supramolecular interactions between the perfluorophenyl moieties of FcF_10_ and the difluorophenyl-based FIC end groups of Y6, leading to a stabilized morphology of the active layer and enhanced thermal stability.

**Figure 8:**
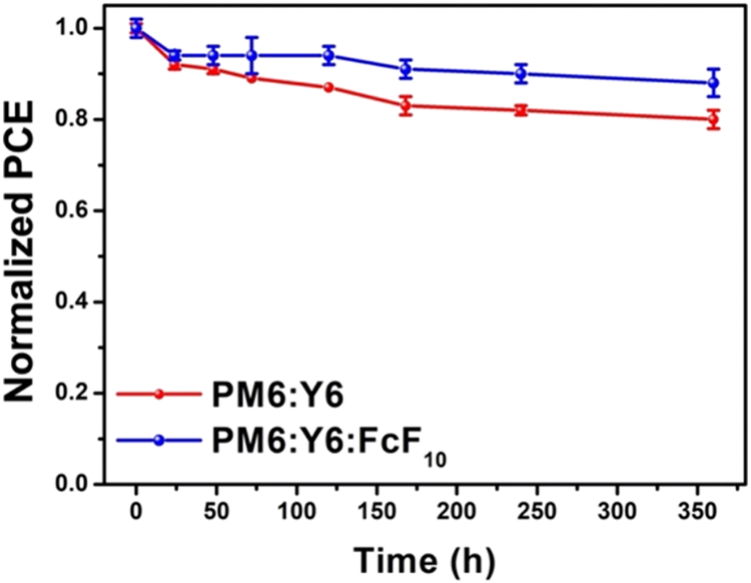
Normalized performance of the PM6:Y6 and PM6:Y6:FcF_10_ devices as a function of time at 85 °C.

Compared to other additives, FcF_10_ exhibits characteristics similar to the non-volatile additive BF7 due to the presence of perfluorophenyl units that engage in strong non-covalent interactions with fluorinated acceptors Y6. Unlike volatile ferrocene (Fc), which evaporates during annealing, FcF_10_ remains in the active layer. Its ferrocene-based core introduces a three-dimensional structure that not only promotes crystalline packing but also serves as a spacer between Y6 domains, regulating morphology and enhancing its long-term stability.

## Conclusions

In conclusion, this study demonstrates that the incorporation of FcF_10_ as a non-volatile solid additive significantly enhances both the performance and thermal stability of PM6:Y6-based OSCs. The PM6:Y6 device with 3.75 wt % of FcF_10_ achieved a higher PCE of 17.00%, with a *V*_oc_ of 0.85 V, a *J*_sc_ of 7.35 mA cm^–2^, and an FF of 73.29%, outperforming the reference device with a PCE of 15.4%. These improvements are attributed to the formation of a favorable active layer morphology, which enhances exciton dissociation and charge transport while reducing bimolecular and trap-assisted recombination. The FcF_10_ additive facilitates non-covalent interactions with Y6, such as F···F, F···π, and π···π interactions between the Cp and C_6_F_5_ rings in FcF_10_ and the FIC end groups in Y6. These supramolecular interactions improve molecular stacking and crystallinity within the Y6 domain, as evidenced by red-shifted Y6 absorption, reduced π–π stacking *d*-spacing, and increased coherence lengths of Y6, corroborated by UV–Vis spectroscopy, DSC analysis, GIWAXS, and AFM measurements. Furthermore, thermal stability tests at 85 °C demonstrate the ability of FcF_10_ in maintaining device performance, with an 88% retention of the initial PCE after prolonged thermal annealing.

## Supplementary Material





## Notes

NMR, device fabrication, SCLC data, 1D GIWAXS, and single-crystal parameters (PDF)
Fc-10F (CIF)
