# Supplementary material for: Perfluorophenyl-Incorporated Ferrocene: A Non-Volatile Solid Additive for Boosting Efficiency and Stability in Organic Solar Cells
Source: ACS Appl Mater Interfaces. 2025 May 20;17(22):32722–31. doi: 10.1021/acsami.5c04989 (PMC12147079; doi:10.1021/acsami.5c04989)
Supplement: Supplementary file 1 [file am5c04989_si_001.pdf]

Supporting Information

Perfluorophenyl-Incorporated Ferrocene: A Non-Volatile Solid Additive for Boosting Efficiency and Stability in Organic Solar Cells

Chia-Lin Tsai,[‡a] *Han-Cheng Lu,*[‡a] *Chi-Chun Tseng,*[a] *Yung-Jing Xue,*[a] *Kai-En Hung,*[a] *Chia-Shing Wu,*[c] *Chia-Chih Chang,* [ab] *Chain-Shu Hsu,* [ab]*Katarina Gugujonovic,*[e] *Markus Clark Scharber,*[e] *Fong-Yi Cao*[d]* *and Yen-Ju Cheng**[ab]

[a]Department of Applied Chemistry, National Yang Ming Chiao Tung University, 1001 University Road, Hsinchu, Taiwan 30010.

[b]Center for Emergent Functional Matter Science, National Yang Ming Chiao Tung University, 1001 University Road, Hsinchu, Taiwan 30010.

[c]Taiwan Space Agency, 8F, 9 Prosperity 1st Road, Hsinchu Science Park, Hsinchu, Taiwan *300091*

[d]Department of Chemistry, National Changhua University of Education, Changhua City, Taiwan 50007

[e]Institute of Physical Chemistry and Linz Institute of Organic Solar Cells (LIOS), Johannes Kepler University Linz, Altenbergerstrasse 69, 4040 Linz, Austria.

Corresponding Author

Yen-Ju Cheng

E-mail: yjcheng@nycu.edu.tw

Fong-Yi Cao

E-mail: fycao@cc.ncue.edu.tw

Table of Contents

1. Device fabrication process and J-V^2 plot of SCLC ………………………………………S3-S4

2. The crystallographic data of FcF_{10}. …………………………………….....................……S5

3. 1D line-cut profiles of GIWAXS diffraction pattern …...……………………….…………S7

4. Reference…………………………………………………………………………………...S8

5. Nuclear Magnetic Resonance Spectroscopy (NMR)……………………………………..S9

1. Device fabrication process and *J*-*V*^{2} plot of SCLC

We employed an inverted device configuration: ITO/ZnO/Active Layer/MoO_3/Ag. The ITO glass substrates were sequentially cleaned using detergent, deionized water, acetone, and isopropyl alcohol under ultrasonic treatment, followed by UV-O_3 cleaning. The ZnO precursor was prepared by dissolving zinc acetate dihydrate ($Zn(CH_3COO)_2 \cdot 2H_2O$, Aldrich, 99.9%, 1 g) and ethanolamine ($NH_2CH_2CH_2OH$, Aldrich, 99.5%, 0.28 g) in 2-methoxyethanol ($CH_3OCH_2CH_2OH$, Aldrich, 99.8%, 10 mL) with vigorous stirring for 24 hours. This solution was then spin-coated onto a pre-cleaned ITO substrate, followed by baking at 170 °C in air for 30 minutes. The blend solution of the photoactive layer consisted of PM6:Y6 (1:1.2 by weight) in chloroform at a total concentration of 17.6 mg/mL, with the additive of 0.6 vol% CN and varying FcF_{10} content (from 0.625 wt% to 5 wt%). This solution was spin-coated onto the ZnO layer to form the active layer in the glovebox, which was subsequently thermally annealed at 100 °C for 13 min. Shadow masks were then applied to define the active area (0.04 mm^2) before transferring the devices to a thermal evaporator. The hole transporting layer and anode, composed of MoO_3 (7 nm) and silver (150 nm) layers, were sequentially deposited onto the active layer through thermal evaporation. Solar cell measurements were conducted at an air-conditioned temperature of approximately 25 °C using an Oriel Xenon lamp (450 W) with an AM1.5 filter as the solar simulator. The light intensity was calibrated to 100 mW/cm^2 using a standardized silicon solar cell. Device parameters for the PM6:Y6 devices with various FcF_{10} weight contents are shown in **Table S1**.

Table S1. The device parameters for the PM6:Y6-based devices with different FcF_{10} weight content.

FcF_{10}	V_{oc} (V)	J_{sc} (mA/cm^2)	$J_{sc}^{cal.}$ (mA/cm^2)	FF (%)	PCE (%)	R_s (ohm)	R_{sh} (kohm)
NA	0.84	25.50	25.42	71.74	15.34	122.74	13.80
0.625 w%	0.84	26.07	24.81	72.80	16.09	90.51	16.30
1.25 w%	0.84	26.37	25.09	71.86	15.98	123.74	19.45
2.5 w%	0.84	26.59	25.11	73.25	16.35	88.07	21.08
3.75 w%	0.85	27.35	26.72	73.29	17.00	102.58	43.57
5 w%	0.83	25.88	25.00	72.36	15.73	114.53	44.30

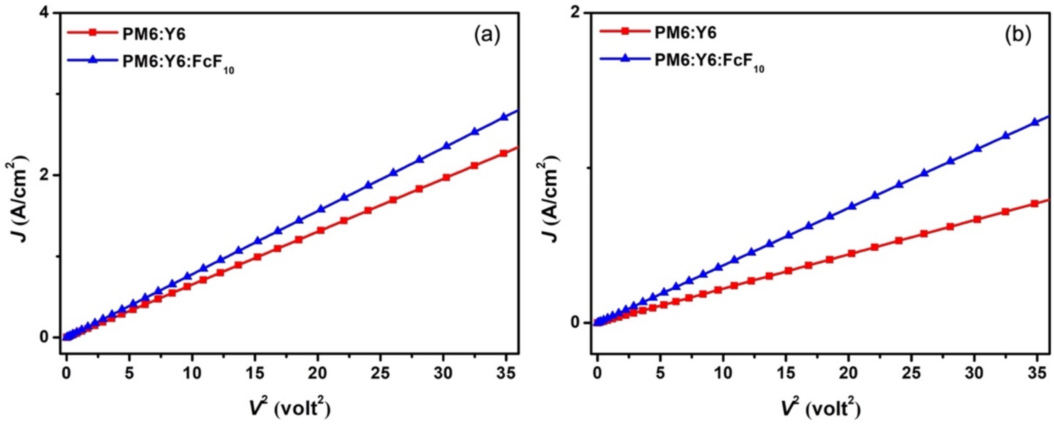

Figure S1. J-V^2 plot of SCLC: (a) hole-only devices (b) electron-only devices

2. The crystallographic data of FcF_{10}

An orange single crystal of FcF_{10} was mounted on a CryoLoop using Parabar 10312 oil for single-crystal X-ray diffraction analysis at −123 °C. The crystal dimensions were 0.240 × 0.170 × 0.130 mm³. Data collection was performed in-house on a Bruker D8 Venture diffractometer equipped with a Mo-target microfocus X-ray generator (Kα = 0.71073 Å) and a PHOTON-II CMOS detector. Temperature control was achieved using a nitrogen flow system (Oxford Cryosystems, 800+ series). Data collection, cell refinement, and integration were performed using the Bruker SAINT v8.40B[1] software package, employing a narrow-frame algorithm. Absorption corrections were applied using the Multi-Scan method via SADABS software[2]. The molecular structure was solved using SHELXT and refined using SHELXL-2019/1 with anisotropic full-matrix least-squares refinement on F^2 [3]. All calculations were conducted using the APEX4 software suite [4]. The crystallographic data for FcF_{10} are summarized in **Table S2** in the Supporting Information.

Table S2. The crystallographic data of FcF_{10}.

CCDC number	2400677
Empirical formula	$C_{24}H_8F_{10}FeO_4$
Formula weight	606.15
Temperature	150(2) K
Wavelength	0.71073 Å
Crystal system	Monoclinic
Space group	$P2_1/n$
Unit cell dimensions	a = 11.7215(4) Å α = 90°<br>b = 7.6145(2) Å β = 104.8952(13)°<br>c = 12.2369(3) Å γ = 90°
Volume	1055.48(5) Å3
Z	2
Density (calculated)	1.907 Mg/m^3
Absorption coefficient	0.837 mm^{-1}
F(000)	600
Crystal size	0.240 × 0.170 × 0.130 mm^3
θ range for data collection	3.182 to 27.918°
Index ranges	−15 <= h <= 15, −10 <= k <= 10, −16 <= l <= 16

Reflections collected	21170
Independent reflections	2504 [R(int) = 0.0423]
Completeness to θ = 25.242°	99.3%
Refinement method	Full-matrix least-squares on F^2
Data/restraints/parameters	2504/0/178
Goodness-of-fit on F^2	1.024
Final R indices [I>2sigma(I)]	R1 = 0.0257, wR2 = 0.0771
R indices (all data)	R1 = 0.0290, wR2 = 0.0798
Largest diff. peak and hole	0.324d −0.243 e.$Å^{-3}$

3. 1D line-cut profiles of GIWAXS diffraction patterns

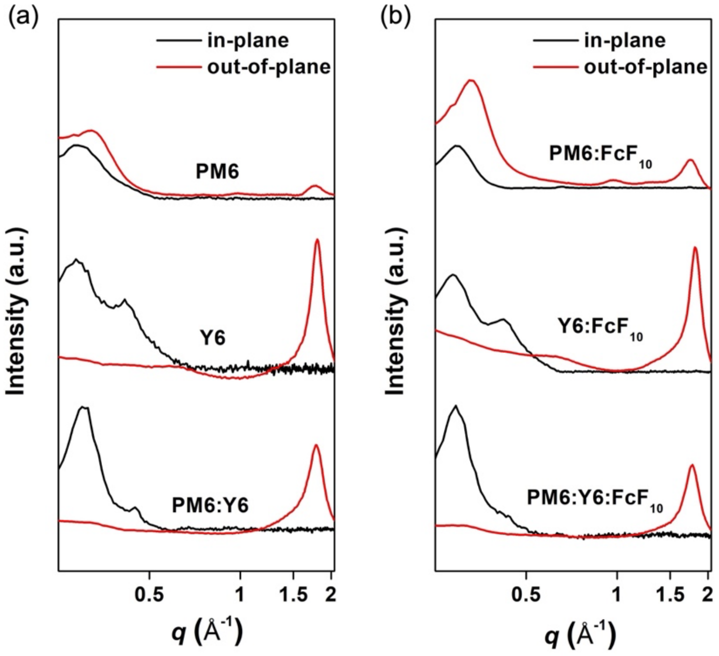

Figure S2. 1D line-cut profiles of GIWAXS diffraction patterns of the (a) PM6, Y6 and PM6:Y6; (b) PM6:FcF_{10}, Y6:FcF_{10} and PM6:Y6:FcF_{10} thin films.

4. References:

1. SAINT v8.40B; Bruker AXS GmbH, Karlsruhe, Germany, **2019**.
2. Krause, L.; Herbst-Irmer, R.; Sheldrick, G. M.; Stalke, D. *J. Appl. Crystallogr.* **2015**, *48*, 3–10.
3. Sheldrick, G. M. *Acta Crystallogr.* **2015**, *C71*, 3–8.
4. APEX4 v2021.10-0; Bruker AXS GmbH, Karlsruhe, Germany, **2021**.

5. Nuclear Magnetic Resonance Spectroscopy (NMR)

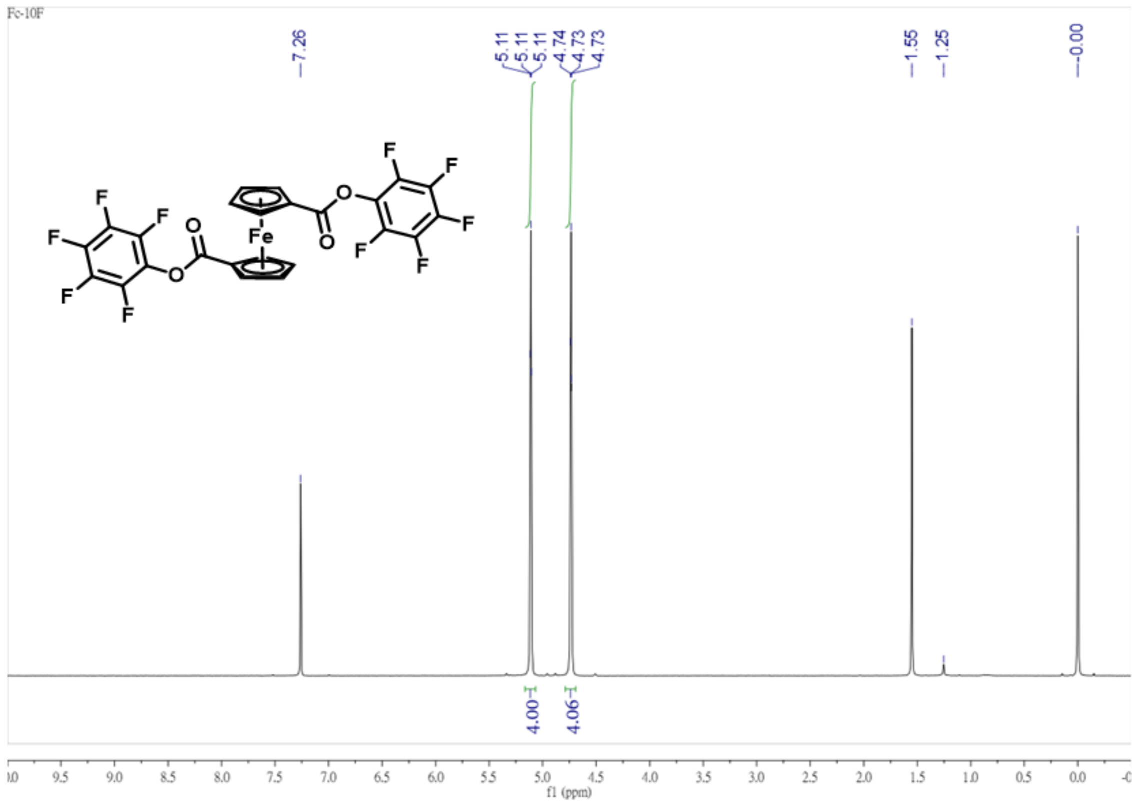

Figure S3. ^{1}H NMR spectrum of FcF_{10}

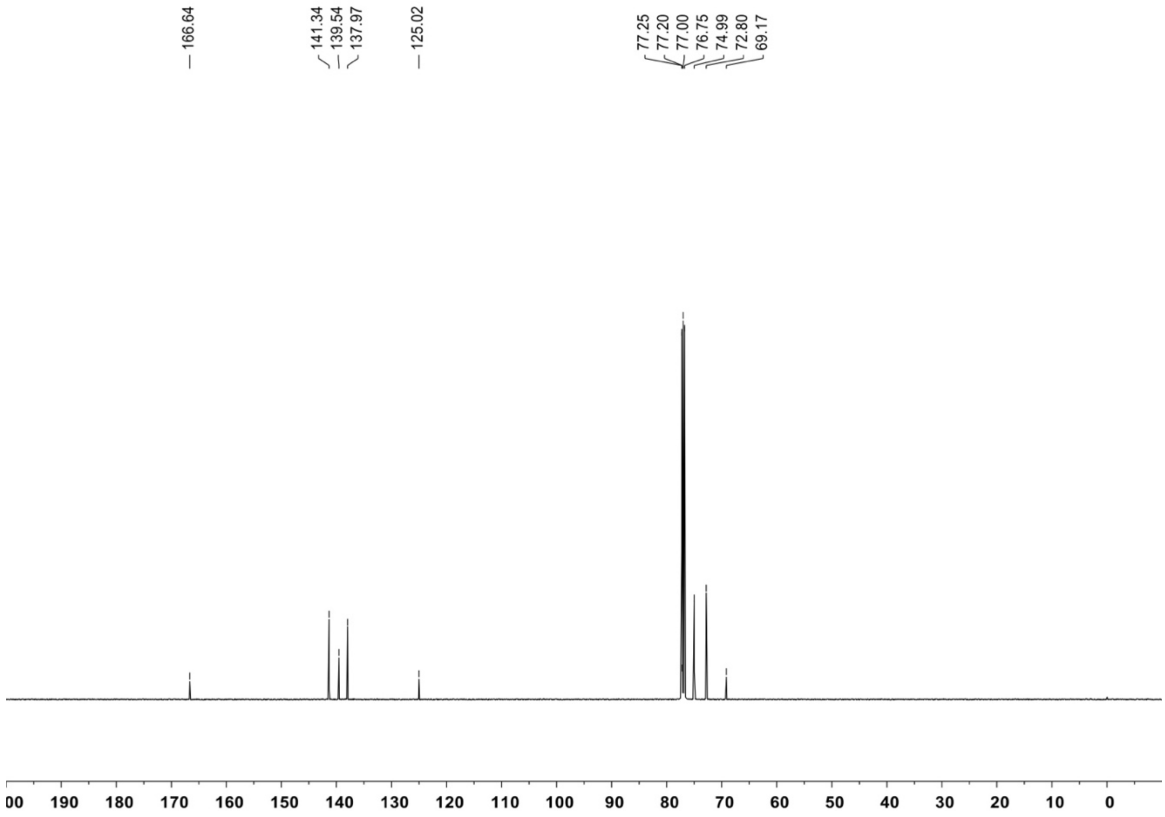

Figure S4. ^{13}C NMR spectrum of FcF_{10}
